# Defining a Therapeutic Window of Opportunity in Alopecia Areata: Predictors of Early Response to Baricitinib

**DOI:** 10.3390/jcm14207312

**Published:** 2025-10-16

**Authors:** Daniel Muñoz-Barba, Carmen García-Moronta, Alberto Soto-Moreno, Manuel Sánchez-Díaz, Salvador Arias-Santiago

**Affiliations:** 1 Dermatology Unit, Hospital Universitario Virgen de las Nieves, 18012 Granada, Spain; danielmb00w@gmail.com (D.M.-B.); carmengmoronta@gmail.com (C.G.-M.); albertoide@aol.es (A.S.-M.); salvadorarias@ugr.es (S.A.-S.); 2 Dermatology Department, School of Medicine, University of Granada, 18016 Granada, Spain; 3 Institute of Biosanitary Research IBS, 18012 Granada, Spain; 4 Trichology Clinic, Hospital Universitario Virgen de las Nieves, 18012 Granada, Spain

**Keywords:** alopecia areata, baricitinib, early responders, Janus kinase inhibitors

## Abstract

**Background/Objectives**: Baricitinib, a selective Janus kinase (JAK) 1 and 2 inhibitor, has recently emerged as a therapeutic option for patients with severe alopecia areata (AA). The aim of this study was to identify clinical and biological predictors of early therapeutic response to baricitinib in patients with AA in real-world clinical practice. **Methods**: A retrospective cohort study was conducted including patients with AA initiating baricitinib between January 2022 and January 2025. Patients were stratified into early responders and non-early responders. Univariate and multivariate logistic regression analyses were performed to assess factors independently associated with early therapeutic response. **Results**: A total of 44 patients with AA treated with baricitinib were included, the majority being female (65.9%, 29/44), with a mean age of 37.3 years (SD 16.1). Early responders accounted for 34.1% (15/44) of the cohort. In multivariate analysis, early response to baricitinib was independently associated with a lower baseline Severity of Alopecia Tool (SALT) score, shorter disease duration, and elevated erythrocyte sedimentation rate (ESR) at baseline (*p* < 0.05). Receiver Operating Characteristic (ROC) curve analyses were performed to determine optimal thresholds for predicting early therapeutic response: ESR ≥ 9 mm/h, baseline SALT score ≤ 60%, and disease duration ≤ 7 years. **Conclusions**: Baseline stratification using easily obtainable clinical and laboratory parameters may help identify patients most likely to benefit from initiating treatment with baricitinib. Our findings support the existence of a therapeutic window of opportunity in AA, particularly in patients with lower disease burden, shorter disease duration, and elevated ESR values.

## 1. Introduction

Alopecia areata (AA) is an autoimmune disease of the hair follicle that causes non-scarring alopecia. The onset of the disease covers a wide age range and can vary from the appearance of small patches of alopecia on the scalp to a total absence of body hair. Due to the resulting disfigurement and unpredictable course, AA significantly reduces quality of life [1,2]. There are currently several treatment alternatives, some with very promising results, such as Janus kinase inhibitors (JAKi) [3,4].

JAKis have been widely used in and are approved for other autoimmune and inflammatory conditions [5]. The approval of baricitinib, a JAKi targeting JAK1 and JAK2, by the FDA in 2022 has significantly transformed the therapeutic approach to AA. Th1 cytokines, including interferon-gamma (IFN-γ) and interleukin (IL)-15, activate the JAK–STAT pathway, triggering inflammation and an autoimmune assault on hair follicles. By inhibiting this signalling cascade, JAKis effectively suppress pathogenic immune responses, offering a promising treatment for AA [6,7].

Since then, several studies in real clinical practice have reported the effectiveness of baricitinib in the treatment of this disease in terms of hair regrowth measured by the Severity of Alopecia Tool (SALT) index [8,9,10,11,12]. However, to date, there are few studies assessing the presence of predictors of response to treatment with baricitinib [13,14].

Identifying predictors of early response to baricitinib in patients with AA may facilitate more individualised treatment, improve adherence through realistic expectation setting and reduce early treatment discontinuation. In our clinical experience, one of the most common questions posed by patients initiating JAKi therapy is when they can expect to see hair regrowth. The ability to anticipate early responders may therefore allow clinicians to offer more precise, evidence-based guidance regarding treatment timelines, helping to reduce uncertainty, build patient trust, and encourage sustained therapeutic engagement. This retrospective study aims to investigate such predictive factors in real-world settings, providing insights that may refine current stratification strategies and support more personalised therapeutic decision-making.

## 2. Materials and Methods

### 2.1. Study Design

A retrospective observational cohort study was conducted to evaluate real-world predictors of early clinical response to baricitinib in patients diagnosed with AA who were candidates for initiation of JAKi therapy. Eligible patients were recruited over a three-year period, between January 2022 and January 2025, at a tertiary-level Spanish dermatology department within a dedicated trichology consultation unit. All patients included in the study initiated treatment with baricitinib as part of routine clinical care and were monitored every 3 months during at least 12 months follow-up.

### 2.2. Inclusion Criteria

Participants were required to fulfil the following inclusion criteria: (a) diagnosis of AA of at least six months’ duration through clinical and trichoscopic evaluation performed by experienced dermatologists; (b) eligibility for systemic JAKi therapy: in Spain, the public healthcare system provides reimbursement for this treatment in patients with severe AA who present with a SALT score > 50% and have shown no response to therapy with oral dexamethasone pulses or conventional immunosuppressive agents after a minimum of six months; (c) age ≥ 18 years; (d) capacity and willingness to attend scheduled follow-up assessments; (e) provision of written informed consent. Patients who had an insufficient response to previous treatment with JAKi, defined as a SALT score > 20% after at least six months of treatment, or the occurrence of adverse events due to this drug, were also included in the study.

### 2.3. Exclusion Criteria

Exclusion criteria were: (a) withdrawal of consent or refusal to participate; (b) presence of haematological, biochemical, or serological abnormalities contraindicating the initiation of JAKi therapy; (c) history or active diagnosis of malignant neoplasms.

### 2.4. Ethical Considerations

The study protocol received approval from the institutional Research Ethics Committee (code: SICEIA-2025-000370) and was conducted in accordance with the ethical standards set forth in the Declaration of Helsinki and relevant national legislation regarding biomedical research involving human subjects.

### 2.5. Study Variables

#### 2.5.1. Main Variables

I. Severity of Alopecia Tool (SALT): This metric served as an objective gauge of the severity of the disease. It indicates the proportion of the scalp impacted by AA [15].
II. Type of response to baricitinib treatment: Patients were classified into two categories according to classifications used in most recent studies.
  i. Early responders (satisfied the following three conditions):
    a. The attainment of at least a 30% relative improvement in the SALT score within the first 3 months of initiating baricitinib therapy, commonly referred to as achieving a SALT_30_ response [16].
    b. The achievement of a SALT score < 20%, corresponding to a clinically meaningful reduction in scalp involvement [7], within the first 6 months of treatment.
    c. The maintenance of this therapeutic improvement without relapse or deterioration throughout the 12-month follow-up period.
  ii. Non-early responders (satisfied at least one of the following three conditions):
    a. The absence of a ≥30% relative improvement in the SALT score within the first 3 months after initiating baricitinib therapy.
    b. The inability to reach a SALT score < 20% within the first 6 months of treatment.
    c. The failure to maintain a stable therapeutic response throughout the 12-month follow-up period, either due to clinical relapse, secondary loss of efficacy, or fluctuating disease activity that prevented sustained improvement.
III. Eyebrow and Eyelash Involvement: To assess involvement in both areas, a numerical scale ranging from 0 to 3 was employed: 0 indicates complete hair loss; 1 corresponds to a loss of more than 50% of hair density with interspersed alopecic patches; 2 represents a loss of less than 50% without interspersed alopecic areas; 3 denotes normal hair density in both eyebrows and eyelashes.
IV. Potential predictors of early response: Several clinical, analytical and socio-demographic data were considered as potential predictors of early response (13):
    i. Sex, age of onset and disease duration.
   ii. AA pattern and previous treatments.
  iii. Analytical data: As observed for other diseases, hemogram parameters were considered as potential predictors of response:
    a. Neutrophil-to-Lymphocyte ratio (NLR): Value calculated by dividing the total neutrophil count by the total lymphocyte count [17].
    b. Systemic Immune Inflammatory Index (SIII): Index calculated from the following calculation: platelet count × neutrophil–lymphocyte ratio [18].
    c. Erythrocyte Sedimentation Ratio (ESR): Laboratory test that measures the rate at which red blood cells settle at the bottom of a vertical test tube containing anticoagulated blood. The result is expressed in millimetres per hour (mm/h) [19].
    d. C-Reactive Protein (CRP): Acute-phase reactant produced predominantly by hepatocytes in response to pro-inflammatory cytokines. CRP levels may be elevated in various autoimmune and inflammatory conditions, including AA [20].
V. Dermatology Life Quality Index (DLQI): This questionnaire is a validated tool used to assess the overall impact of dermatological conditions on quality of life in individuals aged 16 years or older. It consists of 10 items, each scored on a 4-point Likert scale from 0 (no impact) to 3 (very significant impact). The questionnaire evaluates how the skin condition has affected the patient’s daily life during the previous week [21].

#### 2.5.2. Secondary Variables

Socio-demographic data, comorbidities associated with AA and safety data were also collected at baseline and during follow-up.

### 2.6. Statistical Analysis

Descriptive statistics were used to characterise the study population. Continuous variables were expressed as means with standard deviations (SDs) and categorical variables as absolute and relative frequencies. The Shapiro–Wilk test was used to assess normality. Comparisons between early responders and non-responders were conducted using χ^2^ or Fisher’s exact test for categorical data and Student’s *t*-test or the Wilcoxon–Mann–Whitney U test for continuous variables, as appropriate. Univariable linear regression was employed to explore associations between continuous predictors and treatment response, and odds ratios (ORs) with 95% confidence intervals (CIs) were derived. Variables with *p* < 0.10 in univariate analyses were entered into a multivariable logistic regression model to determine independent predictors of early response. A two-sided *p*-value < 0.05 was considered statistically significant. Receiver operating characteristic (ROC) tables were constructed to assess optimal cut-off points of predicting variables. All analyses were performed using JMP^®^ statistical software, version 14.1.0 (SAS Institute Inc., Cary, NC, USA).

## 3. Results

### 3.1. Socio-Demographic and Clinical Characteristics of the Sample

A total of 44 patients diagnosed with AA and treated with baricitinib were included in the analysis. Female patients constituted 65.9% of the cohort, resulting in a female-to-male ratio of approximately 2:1 (29:15). The mean age was 37.3 ± 16.1 years. A positive family history of AA was reported in 9.1% (4/44) of cases. Autoimmune comorbidities were present in 36.0% of the cohort, with hypothyroidism being the most frequent (20.5%, 9/44). The mean disease duration prior to initiating baricitinib was 10.68 ± 10.24 years, while the average age of onset was 24.8 ± 15.94 years. Total or universal forms of AA were documented in 34.1% (15/44) of patients, and eyebrow and/or eyelash involvement was observed in 52.3% (23/44). The most common clinical subtype was multifocal AA, present in 65.9% (29/44). The baseline SALT score was 67.16 ± 32.52%. All patients had previously received both topical and systemic corticosteroids. Additionally, 34.1% (15/44) had been treated with conventional systemic immunosuppressants, and 22.0% (10/44) had received tofacitinib. Regarding treatment outcome, 34.1% (15/44) were classified as early responders to baricitinib according to established criteria. A detailed distribution of these characteristics is shown in Table 1.

### 3.2. Effectiveness and Safety of Baricitinib According to Response Pattern

**Figure 1:**
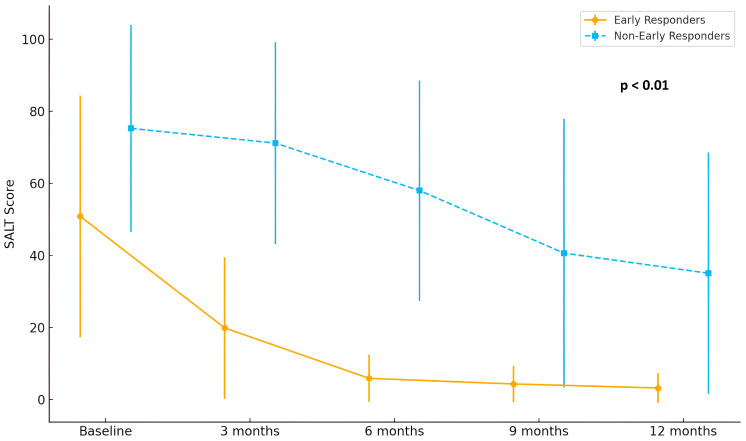
Longitudinal evolution of SALT score during baricitinib treatment in patients with AA. A more pronounced and rapid reduction in SALT score was observed among early responders (*n* = 15) compared to non-early responders (*n* = 29), particularly during the first 6 months of treatment (*p* < 0.01).

As expected based on the predefined classification criteria, early responders exhibited significantly greater reductions in SALT scores at both 3 months (31.66 ± 4.96 vs. 4.48 ± 3.70; *p* < 0.01) and 6 months (45.66 ± 7.39 vs. 18.70 ± 5.85; *p* < 0.01), maintaining a superior clinical response throughout the 12-month treatment period. Detailed SALT evolution is shown in Figure 1.

**Figure 2:**
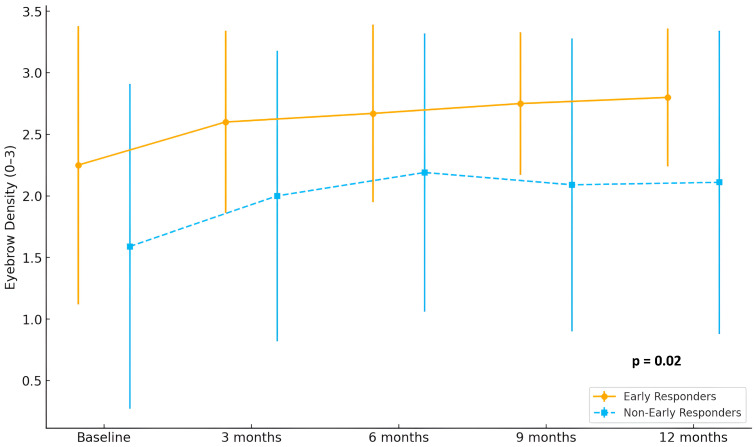
Evolution of eyebrow density scores (range 0–3) in early responders (*n* = 15) and non-early responders (*n* = 29) during 12 months of treatment with baricitinib.

**Figure 3:**
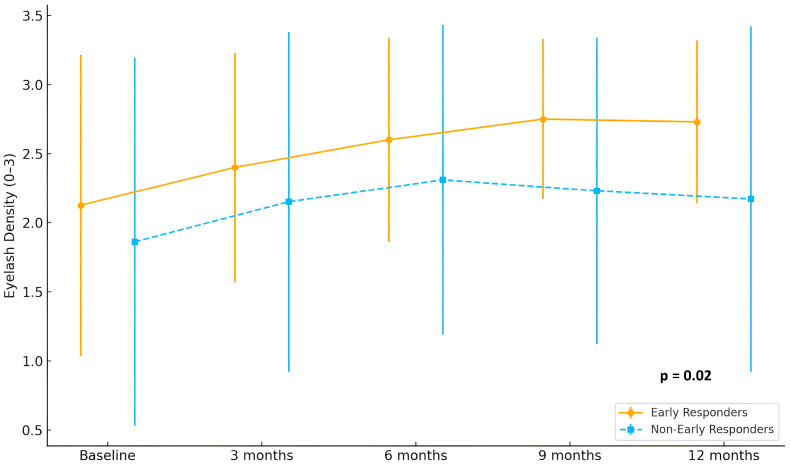
Evolution of eyelash density scores (range 0–3) in early responders (*n* = 15) and non-early responders (*n* = 29) during 12 months of treatment with baricitinib.

In terms of eyebrow involvement, early responders showed a continuous and marked improvement in hair density, with mean scores rising from 2.2 at baseline to 2.9 at 12 months. In contrast, non-early responders demonstrated only a modest increase, from 1.6 to approximately 2.1 (*p* = 0.02). A comparable pattern was observed in eyelash regrowth, where early responders reached mean scores above 2.8 at month 12, while non-early responders remained around 2.2 (*p* = 0.02) Detailed eyebrow and eyelash hair regrowth is shown in Figure 2 and Figure 3.

**Figure 4:**
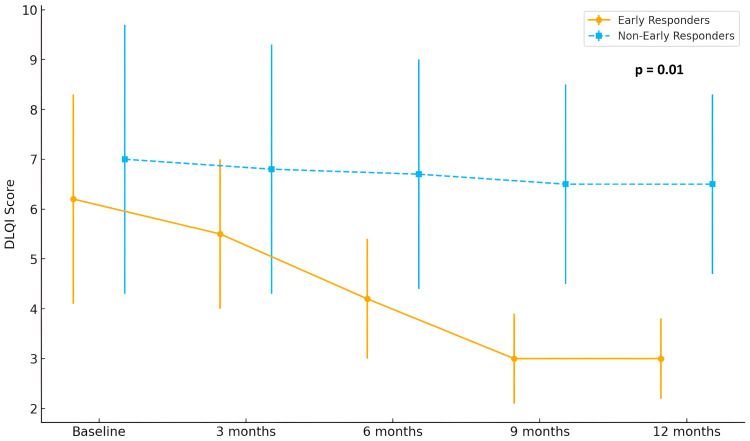
Evolution of Dermatology Life Quality Index (DLQI) scores in early (*n* = 15) and non-early responders (*n* = 29) over 12 months of treatment with baricitinib.

Finally, analysis of patient-reported outcomes revealed a clinically meaningful improvement in quality of life among early responders, with DLQI scores decreasing from 6.2 at baseline to 3.0 at month 12. Meanwhile, DLQI scores in non-early responders remained largely stable over the follow-up period (*p* = 0.01). Detailed improvement in quality of life is shown in Figure 4.

**Figure 5:**
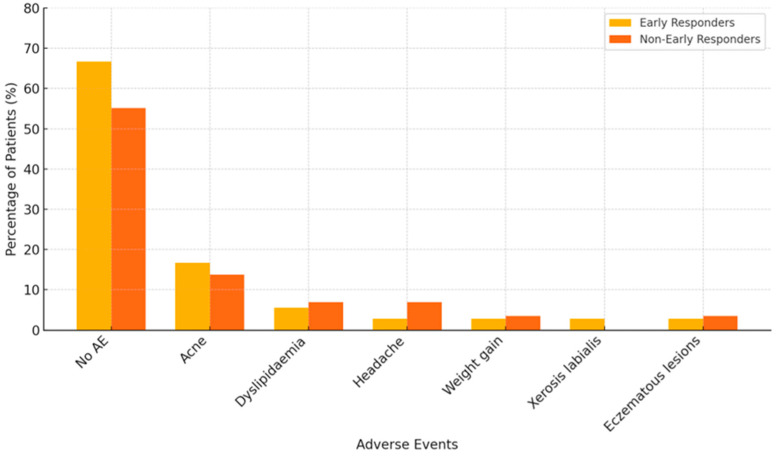
Distribution of adverse events (AE) associated with baricitinib in early responders (*n* = 15) and non-early responders (*n* = 29). Data are expressed as the percentage of patients within each group experiencing the specified adverse event.

The distribution of adverse events (AEs) associated with baricitinib was comparable between early and non-early responders. Most patients in both groups did not report any AEs throughout the 12-month treatment period—66.67% of early responders and 55.17% of non-early responders. Among those who experienced side effects, acne was the most frequently reported in both groups. Other less common AEs included dyslipidaemia, headache, weight gain, xerosis labialis, and eczematous lesions. No serious adverse events or treatment discontinuations due to AEs were observed in either group. Details are shown in Figure 5.

### 3.3. Factors Associated with Early Response to Baricitinib: Univariate Analysis

Univariate analysis between early responders (*n* = 15) and non-early responders (n = 29) to baricitinib treatment revealed several significant differences in baseline clinical and inflammatory characteristics. While no statistically significant differences were observed in sex distribution, family history of AA, tobacco smoking status, hypothyroidism, or prior exposure to immunosuppressive agents or tofacitinib (all *p* values > 0.20), early responders were characterized by a significantly shorter disease duration at baseline (6.10 ± 2.52 vs. 13.14 ± 1.84 years; *p* = 0.02) and a lower initial SALT score (51.53 ± 1.75 vs. 75.24 ± 5.72; *p* = 0.02). Regarding disease phenotype, the prevalence of AAT or AAU was lower among early responders compared to non-responders (20.0% vs. 41.4%), although this difference did not reach statistical significance (*p* = 0.74). On the other hand, the frequency of eyebrow and/or eyelash involvement was comparable between groups (56.8% vs. 62.0%; *p* = 0.57). From an immunoinflammatory perspective, early responders exhibited significantly higher baseline ESR levels compared to non-responders (15.29 ± 2.02 mm/h vs. 9.86 ± 1.45 mm/h; *p* = 0.03), whereas other systemic inflammatory markers did not differ significantly between groups (*p* > 0.10). No significant differences were observed in any of the inflammatory parameters between patients previously treated with tofacitinib and those who had not received any JAKi. Details are shown in Table 2.

### 3.4. Factors Associated with Early Response to Baricitinib: Multivariate Analysis

To identify independent predictors of early response to baricitinib, a multivariate logistic regression analysis was performed, including the three baseline variables that had shown statistical significance in the univariate analysis (disease duration, baseline SALT score, and ESR). Age, gender and the presence of autoimmune comorbidities were included in the analysis to control for possible confounding factors related to physiological changes in ESR. The final model revealed that all three variables were significantly and independently associated with early clinical response. Specifically, shorter disease duration showed a tendency to act as a protective factor (β = –0.21 ± 0.11; *p* = 0.06); lower baseline SALT scores were independently associated with early response (β = –0.03 ± 0.01; *p* = 0.03); and higher baseline ESR values were positively associated with early response (β = 0.12 ± 0.06; *p* = 0.04). Details are shown in Table 3.

### 3.5. Predictive Thresholds for Early Treatment Response to Baricitinib: ROC Curve Analysis

To further explore the discriminative ability of the baseline variables that were independently associated with early therapeutic response in the multivariate analysis, ROC curve analyses were performed. For systemic inflammation, the ROC analysis identified an optimal ESR threshold of 9 mm/h for predicting early response, corresponding to a sensitivity of 66.7%, a specificity of 55.2%, and a Youden’s J index of 0.2184. The area under the curve (AUC) was 0.63. In terms of disease chronicity, a duration of 7 years emerged as the optimal cut-off, achieving a sensitivity of 80.0%, a specificity of 60.7%, and the highest Youden’s J index among the variables tested (0.4071). The AUC was 0.66. Regarding baseline disease severity, a SALT < 60% provided the best balance between sensitivity (66.7%) and specificity (65.5%), with a Youden’s J index of 0.3218 and the highest AUC of the three parameters (0.69). 

## 4. Discussion

### 4.1. Definition of Early Response Pattern

Establishing a robust and clinically meaningful definition of early response is essential. In this study, we adopted a composite definition based on criteria reported in pivotal clinical trials and previous publications. Specifically, we defined early responders as those who achieved a SALT_30_ by month 3, consistent with the endpoints used in the BRAVE-AA1 and BRAVE-AA2 trials of baricitinib [16], as well as other real-world studies [11]. In addition, patients were required to reach a SALT score of <20 by month 6, in accordance with the definitions used in the ALLEGRO Phase 2b/3 and ALLEGRO-LT studies for ritlecitinib [22]. We believe that both relative and absolute measures of improvement are necessary to classify a patient as an early responder. For instance, individuals with AAT or AAU who achieve a SALT_30_ response during the first few months but fail to reach a clinically meaningful threshold (SALT < 20) [7] within the first 6 months of treatment should not be classified as early responders, as this could lead to an overestimation of therapeutic efficacy and unrealistic expectations for patients. Conversely, patients who achieve a SALT < 20 score at month 6 without demonstrating at least a SALT_30_ improvement within the first 3 months should not be considered rapid responders either, as they require a longer period, up to six months, to reach a clinically meaningful outcome. These criteria represent clinically relevant milestones that capture both the onset and durability of treatment response, and they align well with the typical cadence of follow-up assessments in routine dermatological practice, thereby reinforcing their applicability in real-world therapeutic decision-making. Based on this mentioned definition, the proportion of early responders in our real-world cohort was 34.10% (15/44). This proportion is broadly in line with that reported in pivotal clinical trials [16], yet appears higher than rates documented in some real-world studies [11]. This discrepancy may be partly attributed to the clinical profile of our patient population, which was characterized by a lower mean baseline SALT score [13]. Finally, it is worth emphasising that early responders exhibited significantly greater rates of eyebrow and eyelash regrowth, along with more pronounced improvements in health-related quality of life, without being exposed to higher rates of medication-related adverse events. These results are particularly meaningful in a patient population that is known to experience elevated rates of mood disorders compared to the general population [23].

### 4.2. Factors Associated with Early Response to Baricitinib

Secondly, although the clinical efficacy of JAKi in patients with AA is well established, there remains a paucity of data identifying specific clinical characteristics that may guide patient stratification and inform therapeutic decision-making. To date, nearly all available evidence regarding predictors of early response to JAKi in AA has been derived from retrospective analyses [13,14,24].

#### 4.2.1. Baseline SALT Score

A consistent trend in the literature suggests that a lower baseline SALT score is associated with a more favorable therapeutic response, underscoring the importance of disease severity at treatment initiation. Although we also observed that patients with lower baseline SALT scores exhibited a more rapid response, no significant differences were found in early response rates among patients with alopecia areata totalis or universalis (AAT/AAU), in contrast to the findings reported in some previous real-life clinical practice studies [9,11,25].

#### 4.2.2. Disease Duration

Wada-Irimada et al. [13] also identified shorter disease duration as a predictive factor for early clinical improvement, suggesting that early intervention during the disease course may be critical for optimizing the therapeutic response to JAKi. The findings of our study are consistent with these observations and support the concept of initiating treatment during the initial stages of the disease. However, in contrast to the findings of that research group, we did not observe significant differences regarding sex, prior exposure to systemic corticosteroid therapy and eyebrows or eyelashes involvement [13].

#### 4.2.3. Inflammatory Biomarkers

Zhang et al. underscored the potential utility of these biomarkers for stratifying treatment outcomes in AA [14]. Recent evidence has demonstrated that patients with AA exhibit significantly elevated levels of systemic inflammatory biomarkers compared to healthy controls. In particular, the NLR and SII were consistently higher in individuals diagnosed with AA. Furthermore, some of these biomarkers have shown dynamic changes in response to intralesional triamcinolone therapy [26]. However, to date, no studies have specifically evaluated the role of these inflammatory indices in patients with AA undergoing treatment with baricitinib.

Additionally, other previous reports have shown that ESR values are elevated in several immune-mediated dermatological diseases, such as AA, when compared to healthy controls [27]. Nevertheless, recent reviews on the pathophysiology and management of AA state that systemic inflammatory markers, such as ESR, are usually within normal ranges in most patients, unless there is associated systemic autoimmune comorbidity [28,29]. Although the elevation of systemic inflammatory markers is not consistent across all patients with AA [30], their measurement may still hold clinical value. Our findings suggest that elevated ESR values may serve as an early predictor of treatment response in patients with AA. This predictive capacity may reflect the presence of an immunoinflammatory phenotype more amenable to JAK inhibition, supported by previously reported correlations between increased ESR levels and the expression of specific transcription factors within the STAT signaling pathway [31]. Such associations have led to the incorporation of ESR as a marker of therapeutic efficacy in other immune-mediated conditions, notably rheumatoid arthritis [32,33]. Similarly, in dermatological settings, elevated ESR has been linked to higher disease activity and better therapeutic outcomes with biologic agents in disorders such as psoriasis and hidradenitis suppurativa [17,18]. Collectively, these findings underscore the potential of ESR as a cost-effective, widely available biomarker to support clinical decision-making in chronic inflammatory dermatoses, including AA, particularly within the context of targeted therapies. Importantly, no significant intergroup differences were observed in other systemic inflammatory parameters.

### 4.3. Defining the Therapeutic Window of Opportunity

ROC curve analyses were performed to establish clinically relevant cut-off values that may assist in predicting which patients are more likely to achieve an early therapeutic response, and conversely, which patients may require prolonged treatment courses, given the not insignificant proportion of individuals exhibiting a delayed response to the drug [34].

With regard to baseline scalp involvement, it is important to recognize that a substantial proportion of patients with AA present with a rapidly progressive disease course. In many such cases, individuals experience an abrupt transition from patchy hair loss to more severe phenotypes, such as AAT or AAU [35]. Under these circumstances, the SALT score frequently reaches values approaching 100% by the time patients are referred for specialized dermatological evaluation. This clinical scenario poses a significant challenge for implementing early therapeutic strategies. Although there are case series in which the majority of patients had AAT or AAU and yet still had response rates higher than those in the pivotal studies [36], the scientific evidence published to date is consistent with the idea of initiating treatment before these advanced stages of the disease are reached. Therefore, in individuals presenting with multiple alopecic patches, particularly those who have failed to respond to conventional systemic agents, it may be clinically advantageous to consider the initiation of JAKi therapy before progression to complete scalp involvement occurs. In this context, a SALT score threshold of approximately 60% could serve as a pragmatic and actionable cut-off for initiating treatment. This value is clinically meaningful, as it balances disease burden with the need for timely intervention and aligns with the approved indication of baricitinib in patients with severe AA under current regulatory frameworks (SALT > 50). Importantly, this proposed SALT < 60 threshold identifies, within the eligible population, those most likely to achieve an early and sustained therapeutic response. Moreover, the most recent European Expert Consensus Statement on the Systemic Treatment of AA recommends JAKi as the first-line systemic therapy for moderate-severe forms of the disease, defining this group as patients with SALT > 20. This reinforces the notion that the therapeutic window for JAKi may extend beyond the reimbursement thresholds currently applied, supporting a more flexible, patient-centred approach to early intervention.

With regard to disease duration, some authors have identified a disease duration of 4 years as a statistically significant cut-off for distinguishing between good and poor responders [37], while other clinical trials have applied stricter criteria, excluding individuals with disease evolution exceeding 6 years from enrolment [38]. However, relying solely on time-based exclusion criteria may unintentionally limit access to effective therapies for a subset of patients who could still experience substantial clinical benefit. In our cohort, we found that patients with disease durations of up to seven years remained capable of achieving significant and sustained responses to baricitinib. This finding suggests the use of arbitrary time-based restrictions in clinical protocols could deprive certain patients, who might otherwise benefit, from receiving timely and effective treatment. This perspective aligns with previous real-world studies in which treatment response rates have even exceeded those reported in pivotal clinical trials, including among patients with longstanding AA of more than eight years [38]. Such evidence collectively calls for a reassessment of eligibility criteria in both research and clinical practice to ensure that therapeutic decisions are guided by individual patient profiles and clinical markers of disease activity, rather than duration alone.

Finally, our findings suggest that the ESR may have a potential role in the management of AA. On the one hand, patients with baseline ESR values above 9 mm/h appear to be good candidates for initiating therapy, as this parameter may reflect a higher degree of systemic immune activation. In this subgroup, it may also be clinically relevant to screen for associated autoimmune comorbidities; however, in our cohort, elevated ESR was not associated with a higher prevalence of the most commonly linked autoimmune conditions (*p* > 0.10). Similarly, the presence of autoimmune comorbidities did not appear to correlate with improved therapeutic outcomes (*p* > 0.10). On the other hand, serial ESR assessments throughout the course of treatment could serve, alongside clinical examination, as a complementary tool for monitoring therapeutic response, analogous to its routine use in other autoimmune disorders [39].

It should be noted, however, that the predictive value of these markers is likely to be considerably enhanced when assessed in combination rather than individually. This supports a more integrative and personalised approach to treatment stratification, which may ultimately help define a true therapeutic window of opportunity in patients with AA.

### 4.4. Limitations and Strengths

The main limitations of this study include its single-center design, small sample size, and the geographic homogeneity of the enrolled population, which may limit the generalizability of the findings. Moreover, certain clinical features that have been previously proposed as potential predictors of treatment response, such as the ophiasis pattern of AA, were not systematically analyzed [13]. Moreover, the fact that all patients in the cohort had previously received oral corticosteroid therapy precluded the assessment of any potential association between prior corticosteroid exposure and early therapeutic response. Furthermore, the ROC curve analyses yielded only moderate discriminative ability, limiting their applicability as stand-alone decision-making tools in clinical practice. Nevertheless, these readily available markers gain predictive value when assessed in combination, supporting their potential role within integrative, multi-parameter stratification strategies rather than as isolated predictors. Finally, our study relied exclusively on routine clinical and laboratory parameters, selected for their accessibility, low cost, and immediate applicability in daily practice. While this pragmatic approach strengthens the translational value of our findings, it inevitably restricts the depth of mechanistic insight. Future research should therefore incorporate more sophisticated methodologies, including genetic and epigenetic analyses, as well as longitudinal profiling of cytokines and other immune mediators implicated in AA, to refine predictive accuracy and provide a clearer understanding of the biological basis of treatment response.

The present study offers several notable strengths that reinforce both the validity and the clinical applicability of its findings. First, it constitutes one of the few real-world investigations specifically designed to explore early response predictors to baricitinib in patients with AA, a therapeutic area where real-life evidence remains scarce despite growing clinical interest [40]. Second, by incorporating easily obtainable clinical parameters into both multivariate modelling and ROC curve analyses, the study provides practical, data-driven tools to support early therapeutic decision-making in routine clinical settings. Third, the definition of early responder employed here is both rigorous and clinically meaningful, combining relative (SALT_30_ at 3 months) and absolute (SALT < 20 at 6 months) thresholds, thus capturing not only the rapid onset of efficacy but also its durability over time.

## 5. Conclusions

In conclusion, this exploratory study contributes novel insights into the early therapeutic dynamics of baricitinib in AA, emphasizing the importance of baseline clinical and inflammatory characteristics in optimizing patient selection. Our findings support the conceptual existence of a therapeutic window of opportunity, particularly in patients with shorter disease duration (<7 years), lower baseline SALT scores (<60%), and elevated ESR (>9 mm/h), during which initiating baricitinib may yield enhanced and sustained clinical and psychological benefits without compromising its safety profile. While causality cannot be established, these results provide a foundation for more personalized and efficient therapeutic decision-making in AA and warrant confirmation in larger, prospective, and mechanistic studies.

## Figures and Tables

**Table 1:** Socio-demographic and clinical features of patients with AA of the sample.

**Socio-demographic features of the patients with AA** **(N = 44)**			
Age (years)		37.7 ± 16.10	
Gender		Female 65.90% (29/44) Male 34.10% (15/44)	
Familiar history of AA		9.10% (4/44)	
Tobacco smoking		9.10% (4/44)	
AID		36.36% (16/44)	
Hypothyroidism		20.45% (9/44)	
**Clinical characteristics of the patients with AA** **(N = 44)**			
Disease duration (years)	10.68 ± 10.24	Previous treatments to Baricitinib	
Age of debut (years)	24.80 ± 15.94	Topical corticosteroids: 100.00% (44/44)	Intralesional corticosteroids: 75.00% (33/44)
Age at start of Baricitinib (years)	35.36 ± 16.33	Topical minoxidil: 82.80% (36/44)	Oral minoxidil: 52.30% (23/44)
Basal SALT score (%)	67.16 ± 32.52%	Anthralin/diphenciprone: 15.91% (7/44)	Oral corticosteroids: 100.00% (44/44)
Multiple plaques AA (%)	65.90% (29/44)	Immunosuppressive agents: 34.10% (15/44)	Tofacitinib: 22.73% (10/44)
AA Total or Universalis (%)	34.10% (15/44)	Type of response to baricitinib: Early responder: 34.10% (15/44) Gradual/Late responder: 65.90% (29/44)	
Eyebrows/eyelashes	52.27% (23/44)		

AA: Alopecia Areata; AID: Autoimmune Diseases; SALT: Severity of Alopecia Tool.

**Table 2:** Socio-demographic and clinical characteristics of early responders to baricitinib in comparison with other non-early response patient group.

	Early Responder Mean ± SE/% (N = 15)	Non-Early Responder Mean ± SE/% (N = 29)	*p* Value
Gender	Female: 60.00% (9/15) Male: 40.00% (6/15)	Female: 68.96%(20/29) Male: 31.14% (9/29)	0.55
Family history of AA	Yes: 6.67% (1/15) No: 93.33% (14/15)	Yes: 10.34% (3/29) No: 89.66% (26/29)	0.68
Tobacco smoking	Yes: 6.67% (1/15) No: 93.33% (14/15)	Yes: 10.34% (3/29) No: 89.66% (26/29)	0.68
Hypothyroidism	Yes: 13.33% (2/15) No: 83.67% (13/15)	Yes: 27.59% (8/29) No: 52.41% (21/29)	0.28
Age of onset (years)	26.40 ± 4.15	23.9 ± 2.98	0.63
Disease duration (years)	6.10 ± 2.52	13.14 ± 1.84	0.02
Basal SALT score before treatment (%)	51.53 ± 1.75	75.24 ± 5.72	0.02
AA Total or Universalis	Yes: 20.00% (3/15) No: 80.00% (12/15)	Yes: 41.38% (12/29) No: 58.62% (17/29)	0.74
Eyebrows/eyelashes involvement	Yes: 53.33% (8/15) No: 46.67% (7/15)	Yes: 62.02% (18/29) No: 37.93% (11/29)	0.57
Prior immunosuppressive therapy	Yes: 46.67% (7/15) No: 53.33% (8/15)	27.59% (8/29) 72.41% (21/29)	0.21
Prior Tofacitinib therapy	Yes: 26.67% (4/15) No: 73.33% (11/15)	Yes: 20.69% (6/29) No: 79.31% (23/29)	0.65
SALT score reduction after 3 months of treatment (%)	31.66 ± 4.96	4.48 ± 3.70	<0.01
SALT score reduction after 6 months of treatment (%)	45.66 ± 7.39	18.70 ± 5.85	<0.01
SALT score reduction after 9 months of treatment (%)	50.42 ± 9.81	35.60 ± 8.25	0.12
SALT score reduction after 12 months of treatment (%)	49.85 ± 13.98	36.75 ± 10.67	0.46
SIII	596.47 ± 110.37	586.52 ± 79.38	0.94
NLR	2.19 ± 0.37	2.01 ± 0.36	0.70
PLR	129.06 ± 17.90	141.53 ± 18.87	0.57
ESR (mm/h)	15.29 ± 2.02	9.86 ± 1.45	0.03
CRP (mg/L)	6.85 ± 2.97	7.59 ± 2.07	0.84

AA: Alopecia Areata; SALT: Severity of Alopecia Tool; SE: Standard error; SIII: Systemic immune inflammatory index; NLR: Neutrophil/lymphocyte ration; PLR: Platelet/lymphocyte ratio; ESR: Erythrocyte sedimentation rate; CRP: C-Reactive Protein.

**Table 3:** Multivariate analysis to study the factors associated with early response.

	Early Responder (Log Odds Yes/No) R^2^ = 0.32	*p* Value
Disease duration (years)	−0.21 ± 0.11	0.06
Basal SALT (%)	−0.03 ± 0.01	0.03
ESR (mm/h)	0.12 ± 0.06	0.04
Autoimmune comorbidity	0.10 ± 0.46	0.84
Age (years)	−0.02 ± 0.03	0.58
Sex (female)	−0.25 ± 0.46	0.58

SALT: Severity of Alopecia Tool; ESR: Erythrocyte sedimentation rate.

## Data Availability

The data that support the findings of this study are available from the corresponding author upon reasonable request.

## Abbreviations

The following abbreviations are used in this manuscript:

AA	Alopecia Areata
AAT	Alopecia Areata Totalis
AAU	Alopecia Areata Universalis
AUC	Area Under the Curve
CIs	Confidence Intervals
C-RP	C-Reactive Protein
DLQI	Dermatology Life Quality Index
ESR	Erythrocyte Sedimentation Rate
IFN-γ	Interferon-Gamma
IL	Interleukin
JAK	Janus Kinase
JAKi	Janus Kinase Inhibitor
NLR	Neutrophil-to-Lymphocyte Ratio
ORs	Odds Ratios
ROC	Receiver Operating Characteristic
SALT	Severity of Alopecia Tool
SIII	Systemic Immune Inflammatory Index
